# Research on longevity and associated age data of South American anurans: trends, gaps and recommendations

**DOI:** 10.1098/rsos.240973

**Published:** 2024-09-25

**Authors:** Amanda J. C. Brum, Tiago G. dos Santos, Sonia Z. Cechin

**Affiliations:** ^1^ Programa de Pós-graduação em Biodiversidade Animal, Universidade Federal de Santa Maria, 1000 Roraima Avenue, Camobi, Santa Maria, Rio Grande do Sul 97105-900, Brazil; ^2^ Universidade Federal do Pampa, Campus São Gabriel, Avenue Antônio Trilha, 1847, São Gabriel, Rio Grande do Sul 97307-020, Brazil

**Keywords:** amphibia, demography, skeletochronology, review

## Abstract

Longevity is one of the most important characteristics in the life history of organisms. It is directly associated with growth, reproduction and age of sexual maturity. Despite this, little is known about longevity in South American anuran species, a region considered as a hotspot of world diversity. Thus, we carried out a literature review of publications on longevity of South American anurans that used the skeletochronology method to identify the main publication trends, as well as to point out the main information gaps and suggest future directions. We found clear biases when we analysed temporal, spatial and taxonomic patterns in publications on longevity: (i) studies are recent (mostly from 2015 onwards), (ii) bufonids and leptodactylids were the most studied groups, (iii) medium to large species are the most studied, (iv) species with wide geographic distribution, low risk of extinction, (v) the studies are concentrated in Brazil and Argentina, and (vi) most studies are in the Chaco biogeographical sub-region. We suggest that future work prioritizes little explored families and with high species diversity, small-bodied species, with restricted distribution, threatened with extinction, in order to expand the representation of different evolutionary lineages along the biogeographical units of South America.

## 1. Introduction

Life history studies aim at explaining development, growth pattern, reproductive investment and survival of species [1–6]. Longevity is one of the most important age aspects since it is directly associated with growth, reproduction and age of sexual maturity [5,7–9], while providing us with useful clues about species senescence [10].

In the last 50 years, skeletochronology, that is, the determination of age by counting the lines of bone growth of cross-sections of long bones such as phalanges [5,11,12], has been the most used method for determining age in different animal groups. Groups include fossil animals ([13,14]; fish [15], lizards [16,17], snakes [18], chelonians [19,20], birds [11,21], mammals [22,23] and amphibians [24,25]). This non-lethal method has proven highly effective and reliable for determining the age of species [12,24,26].

For amphibians, skeletochronology has been employed not only for age determination of temperate and subtropical regions, in which bone growth lines are formed during the hibernation period [12,24,25,27–30], but also for tropical species that slow down their growth during dry seasons [24,31–33]. Associating the age of organisms with environmental factors helps us understand population dynamics. Once we understand population dynamics, we can better explain population decline and design effective conservation strategies [27,34–36].

Amphibians are currently the most endangered vertebrate group in the world, with approximately 41% of species globally extinct or threatened with extinction [37,38]. South America is home to the greatest diversity of anuran species in the world, with more than 2600 species described and a high rate of endemism [37,39,40]. Despite the high species diversity in the Neotropical region, in which South America is inserted, little is known about age aspects of its species [5]. Therefore, the objective of this study was to carry out a literature review on the availability of longevity for South American anurans to identify the main trends in temporal, spatial and taxonomic publications, as well as to point out the main information gaps and provide suggestions for future directions.

## 2. Material and methods

We carried out a bibliographical survey of scientific articles published in national and international journals that included anuran species that occur in South America. We used the electronic databases Google Scholar (https://scholar.google.com.br/) and Scientific Electronic Library online (Scielo; www.scielo.org) with the following research queries: ‘longevity anura’, ‘age anura’, ‘skeletochronology anura’, demography anura’ and ‘age aspects anura’. We then filtered the articles to select only those with longevity data estimated by the skeletochronology technique for South American anurans. We excluded monographs, academic theses, dissertations and publications in annals. We also did not include the article by Brum *et al*. [41] since it aimed at describing a methodological protocol and used a very low sample number (three individuals).

We extracted the following information from the selected articles: (i) family and species used in the study, (ii) sampling country, (iii) sampling geographic coordinates, (iv) biome in which the species was collected, (v) habitat, distribution and threat category of the studied species, (vi) number of individuals analysed, (vii) average body size (SVL) of the species, (viii) maximum estimated longevity of females, (ix) maximum estimated longevity of males, (x) age of sexual maturity and reproductive potential of specie, and (xi) publication year.

We made a cumulative curve to see how the number of publications increased from 1990 to 2023, the overall period with publications. With this information, we plotted the geographic coordinates extracted from the articles to verify which countries and biogeographical regions (adapted from [42]) have more publications, as well as to detect geographic gaps and possible trends.

We defined the species’ habitat type according to Pincheira-Donoso *et al*. [43], which determines five categories: (a) aquatic: strict divers; (b) semi-aquatic: species that depend on intermittent contact with bodies of water; (c) terrestrial: species predominantly inhabiting the soil; (d) arboreal: species that land on bushes or trees; and (e) fossorial: species that live underground, except during reproductive seasons. We define species distribution as restricted, for species that occur in a single biome, and broad, for species that occur in two biomes or more. The threat category was defined following IUCN categories (https://www.iucnredlist.org/).

## 3. Results

**Figure 1.**
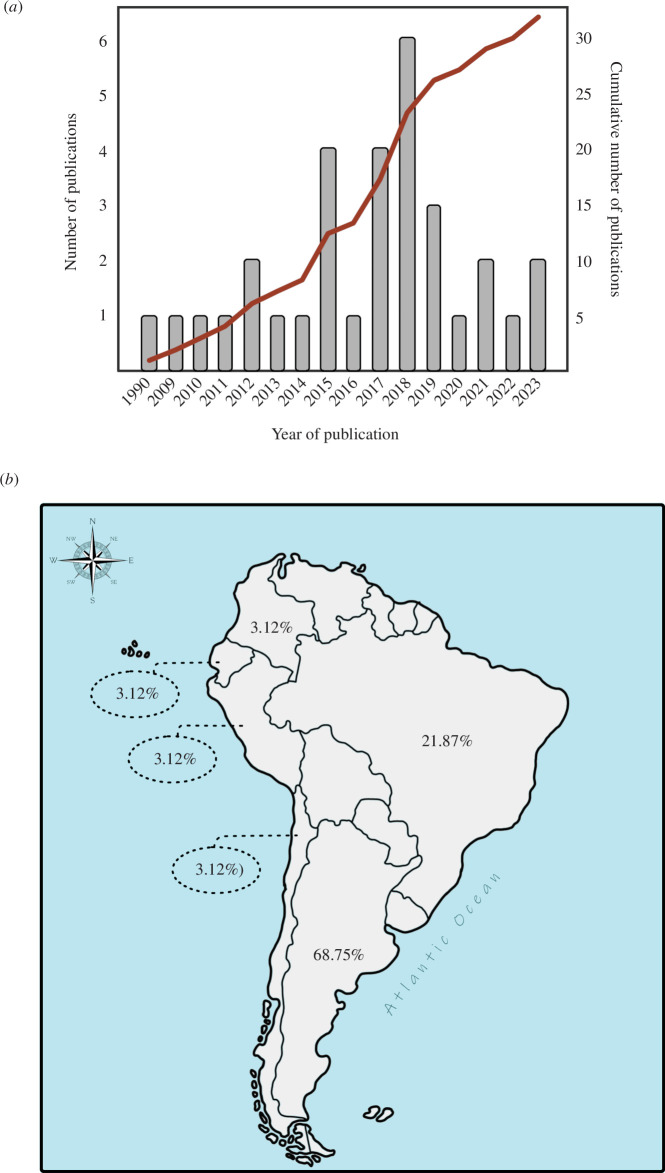
(*a*) Number of articles on skeletochronology of South American anurans published by year. The brown line represents the cumulative number of articles. (*b*) Percentage of studies on the longevity of South American anurans published by country.

We found 32 articles that provided information on South American species using the selected method out of the 160 articles on anuran longevity (electronic supplementary material, S1). Although publications on the longevity of South American anurans began in the 1990s (first publication by [44]), publications only became regular in 2009, with at least one article published per year and peaked from 2015 to 2019 (figure 1*a*). The cumulative curve of articles published from 1990 to 2023 showed an ascending trend, with gradual growth between 1990 and 2014 and a more pronounced increase from 2015 onwards (figure 1*a*). Studies on the longevity of anuran species from South America published in six countries such as Argentina (63.8%) and Brazil (27.7%) together concentrate more than 90% of scientific production in this area (figure 1*b*). Chile, Colombia, Ecuador and Peru hold 2.7% of publications each (figure 1*b*).

**Figure 2.**
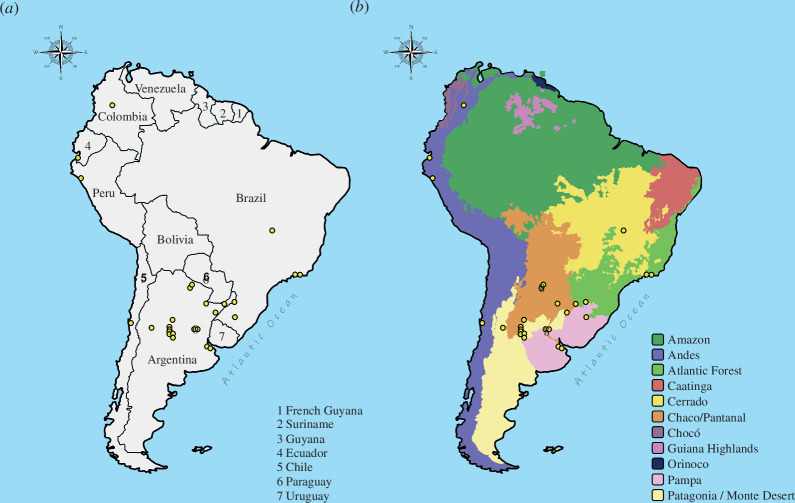
Map of South America, highlighting countries (*a*) and biogeographical regions according to [42] (*b*) Yellow dots represent skeletochronology studies with anurans recovered in our survey.

The 32 articles provided information on the longevity of 36 anuran species from eight families. Argentina provided information on 23 species distributed in six families. Brazil encompassed 10 species distributed in five families. Other countries (i.e. Chile, Colombia, Peru and Ecuador) had only one article, each with information on a single species (table 1, figure 2*a*). Most studies were carried out in the biogeographic region of Chaco/Pantanal. The regions of the Andes, Atlantic Forest and Pampa had three studies carried out in each, and only two and one study were carried out in the Patagonia and Cerrado regions, respectively (figure 2*b*).

**Table 1.** Dataset used in this study. Lat = latitude; Long = longitude. Longevity is expressed in years. Displayed longevity = maximum longevity. All articles cited in the table are shown in the electronic supplementary material, S1.

family	species	longevity		country	coordinates		year	reference
		male	female		lat	long		
Bufonidae	*Atelopus lozanoi*	4	—	Colombia	−4.5128	−73.7382	2012	[31]
Bufonidae	*Atelopus peruensis*	6	—	Peru	−6.9956	−79.6809	2012	[31]
Bufonidae	*Melanophryniscus atroluteus*	7	9	Argentina	−27.4902	−55.6686	2023	[45]
Bufonidae	*Melanophryniscus atroluteus*	6	6	Argentina	−29.0173	−56.9324	2024	[46]
Bufonidae	*Melanophryniscus devincenzii*	7	7	Argentina	−27.4902	−55.6686	2023	[45]
Bufonidae	*Melanophryniscus krauczuki*	5	5	Argentina	−27.4902	−55.6686	2023	[45]
Bufonidae	*Melanophryniscus moreirae*	6	6	Brazil	−22.3849	−44.6782	2015	[47]
Bufonidae	*Rhinella achalensis*	9	11	Argentina	−31.4397	−64.875	2011	[48]
Bufonidae	*Rhinella arenarum*	6	8	Argentina	−34.6083	−58.3712	1990	[44]
Bufonidae	*Rhinella arenarum*	6	4	Argentina	−30.1	−64.4166	2015	[49]
Bufonidae	*Rhinella arenarum*	5	4	Argentina	−33.1238	−64.3490	2018	[50]
Bufonidae	*Rhinella arenarum*	5	5	Argentina	−32.6198	−64.9110	2018	[51]
Bufonidae	*Rhinella ornata*	4	—	Brazil	−22.9222	−43.7763	2019	[52]
Bufonidae	*Rhinella rubescens*	3	3	Brazil	−15.5894	−47.6963	2015	[32]
Bufonidae	*Rhinella diptycha*	5	4	Brazil	−15.5894	−47.6963	2015	[32]
Ceratophryidae	*Ceratophrys cranwelli*	2	—	Argentina	−24.3456	−61.1151	2009	[53]
Ceratophryidae	*Ceratophrys stolzmanni*	3	4	Ecuador	−3.4886	−80.1293	2018	[54]
Ceratophryidae	*Chacophrys pierottii*	5	5	Argentina	−24.9411	−61.4907	2018	[55]
Cycloramphidae	*Thoropa miliaris*	3	—	Brazil	−22.9066	−43.1727	2017	[56]
Hylidae	*Boana cordobae*	5	5	Argentina	−32.6197	−64.9111	2017	[57]
Hylidae	*Boana cordobae*	7	7	Argentina	−32.5931	−64.7108	2018	[58]
Hylidae	*Boana cordobae*	5	6	Argentina	−32.6198	−64.9110	2018	[51]
Hylidae	*Boana pulchella*	5	—	Argentina	−33.1113	−64.3046	2021	[59]
Hylidae	*Nyctimantis siemersi*	5	5	Argentina	−27.4321	−58.7466	2013	[60]
Hylidae	*Scinax fuscovarius*	5	6	Argentina	−29.0173	−56.9324	2023	[46]
Hylodidae	*Crossodactylus schmidti*	6	6	Brazil	−27.2428	−53.9538	2019	[61]
Leptodactylidae	*Leptodactylus bufonius*	4	5	Argentina	−27.4314	−58.7457	2019	[62]
Leptodactylidae	*Leptodactylus latinasus*	6	—	Argentina	−31.7186	−60.2555	2014	[63]
Leptodactylidae	*Leptodactylus latinasus*	3	2	Argentina	−27.4314	−58.7457	2019	[62]
Leptodactylidae	*Leptodactylus luctator*	5	5	Argentina	−31.7047	−60.6672	2017	[64]
Leptodactylidae	*Leptodactylus mystacinus*	7	—	Argentina	−31.7186	−60.2555	2014	[63]
Leptodactylidae	*Physalaemus biligonigerus*	5	4	Argentina	−33.1116	−64.3027	2018	[65]
Leptodactylidae	*Physalaemus cuvieri*	7	7	Brazil	−29.7382	−53.8431	2022	[25]
Leptodactylidae	*Physalaemus fernandezae*	6	6	Argentina	−34.7981	−58.0128	2012	[66]
Leptodactylidae	*Physalaemus riograndensis*	5	5	Brazil	−29.7382	−53.8431	2022	[25]
Leptodactylidae	*Pleurodema cordobae*	4	6	Argentina	−32.3994	−64.9263	2017	[67]
Leptodactylidae	*Pleurodema kriegi*	4	5	Argentina	−31.6127	−64.87472	2017	[67]
Leptodactylidae	*Pleurodema thaul*	5	5	Chile	−30.6666	−71.5166	2010	[68]
Leptodactylidae	*Pseudopaludicola falcipes*	4	5	Brazil	−29.7382	−53.8431	2022	[25]
Microhylidae	*Dermatonotus muelleri*	2	—	Argentina	−24.3456	−61.1151	2009	[53]
Microhylidae	*Dermatonotus muelleri*	5	5	Argentina	−24.9411	−61.4907	2016	[69]
Odontophrynidae	*Odontophrynus americanus*	3	4	Argentina	−31.5166	−67.85	2015	[70]
Odontophrynidae	*Odontophrynus asper*	10	7	Brazil	−29.73828	−53.8431	2020	[29]
Odontophrynidae	*Odontophrynus asper*	6	—	Argentina	−32.7666	−64.2666	2021	[71]
Odontophrynidae	*Odontophrynus asper*	5	7	Argentina	−29.0173	−56.9324	2023	[46]
Odontophrynidae	*Odontophrynus cordobae*	7	—	Argentina	−32.7666	−64.2666	2021	[71]

**Figure 3.**
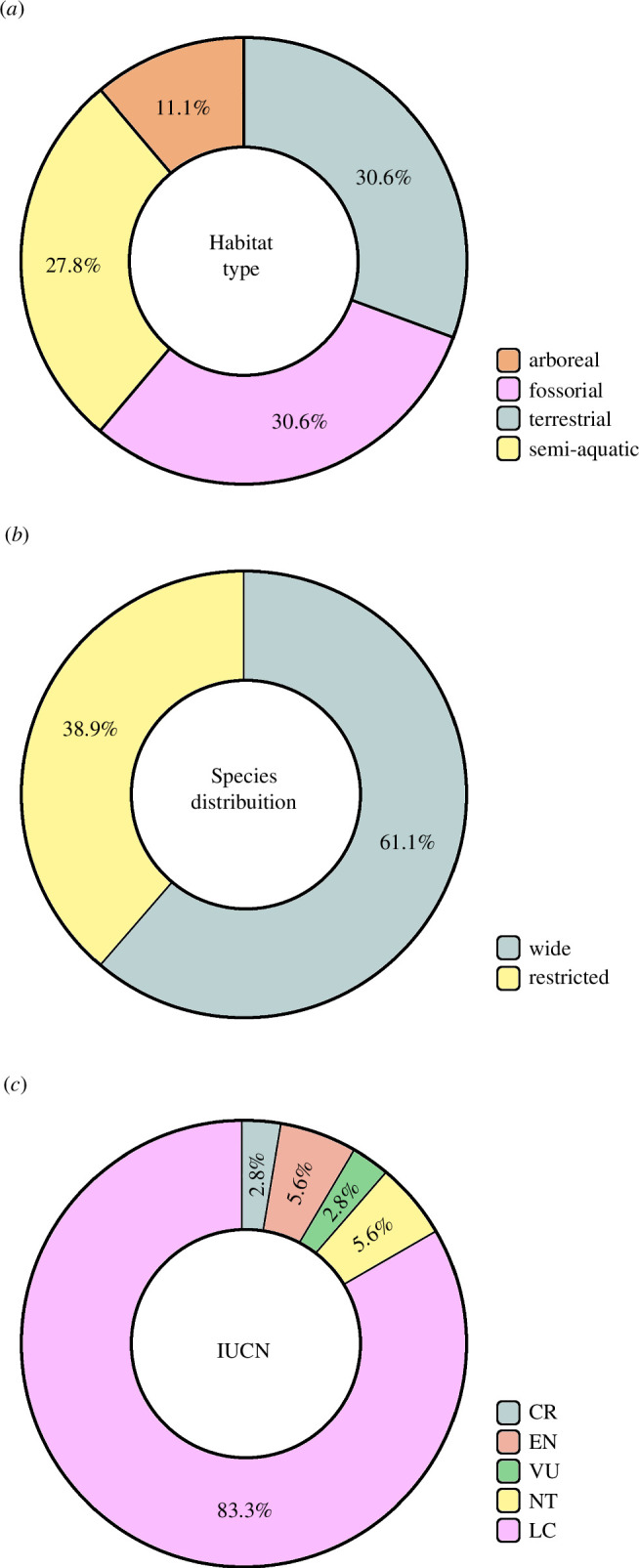
Ecological characteristics of the species, expressed as percentage. (*a*) habitat type, (*b*) species geographic distribution and (*c*) IUCN category.

Species that occupy arboreal habitats (11.1% of studies) are considerably less targeted for longevity studies, when compared with terrestrial, fossorial or semi-aquatic species (30.6%, 30.6% and 27.8% of studies, respectively) (table 2, figure 3*a*). Regarding geographical distribution pattern, more than 60% of the species studied have a wide geographic distribution, while species with a restricted distribution correspond to less than 39% of the studies (table 2, figure 3*b*). More than 80% of South American species used in skeletochronological studies are categorized by the IUCN as Last Concern (LC), followed by the categories Endangered (EN) and Near Threatened (NT), with 5.6% each, and Vulnerable (VU) and Critically Endangered (CR), with 2.8% each (table 2, figure 3*c*).

**Table 2.** Dataset highlighting ecological data of the species. CR = critically endangered, EN = endangered, VU = vulnerable, NT = near threatened and LC = least concern.

family	species	biome	habitat	distribution	IUCN category	reference
Bufonidae	*Atelopus lozanoi*	Andes	terrestrial	restricted	EN	[31]
Bufonidae	*Atelopus peruensis*	Andes	terrestrial	restricted	CR	[31]
Bufonidae	*Melanophryniscus atroluteus*	Chaco/Pantanal	fossorial	wide	LC	[45]
Bufonidae	*Melanophryniscus atroluteus*	Chaco/Pantanal	fossorial	wide	LC	[46]
Bufonidae	*Melanophryniscus devincenzii*	Chaco/Pantanal	fossorial	wide	LC	[45]
Bufonidae	*Melanophryniscus krauczuki*	Chaco/Pantanal	fossorial	restricted	LC	[45]
Bufonidae	*Melanophryniscus moreirae*	Atlantic Forest	fossorial	restricted	NT	[47]
Bufonidae	*Rhinella achalensis*	Chaco/Pantanal	terrestrial	restricted	EN	[48]
Bufonidae	*Rhinella arenarum*	Pampa	terrestrial	wide	LC	[44]
Bufonidae	*Rhinella arenarum*	Pampa	terrestrial	wide	LC	[49]
Bufonidae	*Rhinella arenarum*	Chaco/Pantanal	terrestrial	wide	LC	[49]
Bufonidae	*Rhinella arenarum*	Chaco/Pantanal	terrestrial	wide	LC	[51]
Bufonidae	*Rhinella diptycha*	Cerrado	terrestrial	wide	LC	[32]
Bufonidae	*Rhinella ornata*	Atlantic Forest	terrestrial	wide	LC	[52]
Bufonidae	*Rhinella rubescens*	Cerrado	terrestrial	wide	LC	[32]
Ceratophryidae	*Ceratophrys cranwelli*	Chaco/Pantanal	fossorial	wide	LC	[53]
Ceratophryidae	*Ceratophrys stolzmanni*	Andes	fossorial	restricted	VU	[54]
Ceratophryidae	*Chacophrys pierottii*	Chaco/Pantanal	fossorial	wide	LC	[55]
Cycloramphidae	*Thoropa miliaris*	Atlantic Forest	semi-aquatic	restricted	LC	[56]
Hylidae	*Boana cordobae*	Chaco/Pantanal	arboreal	restricted	LC	[57]
Hylidae	*Boana cordobae*	Chaco/Pantanal	arboreal	restricted	LC	[58]
Hylidae	*Boana cordobae*	Chaco/Pantanal	arboreal	restricted	LC	[51]
Hylidae	*Boana pulchella*	Chaco/Pantanal	arboreal	wide	LC	[59]
Hylidae	*Nyctimantis siemersi*	Chaco/Pantanal	arboreal	restricted	LC	[60]
Hylidae	*Scinax fuscovarius*	Chaco/Pantanal	arboreal	wide	LC	[46]
Hylodidae	*Crossodactylus schmidti*	Atlantic Forest	semi-aquatic	wide	LC	[61]
Leptodactylidae	*Leptodactylus bufonius*	Chaco/Pantanal	semi-aquatic	wide	LC	[62]
Leptodactylidae	*Leptodactylus latinasus*	Chaco/Pantanal	semi-aquatic	wide	LC	[63]
Leptodactylidae	*Leptodactylus latinasus*	Chaco/Pantanal	semi-aquatic	wide	LC	[62]
Leptodactylidae	*Leptodactylus luctator*	Chaco/Pantanal	semi-aquatic	wide	LC	[64]
Leptodactylidae	*Leptodactylus mystacinus*	Chaco/Pantanal	semi-aquatic	wide	LC	[63]
Leptodactylidae	*Physalaemus biligonigerus*	Chaco/Pantanal	semi-aquatic	wide	LC	[65]
Leptodactylidae	*Physalaemus cuvieri*	Pampa	semi-aquatic	wide	LC	[25]
Leptodactylidae	*Physalaemus fernandezae*	Pampa	semi-aquatic	restricted	LC	[66]
Leptodactylidae	*Physalaemus riograndensis*	Pampa	semi-aquatic	wide	LC	[25]
Leptodactylidae	*Pleurodema cordobae*	Patagonia	semi-aquatic	restricted	LC	[67]
Leptodactylidae	*Pleurodema kriegi*	Patagonia	semi-aquatic	restricted	NT	[67]
Leptodactylidae	*Pleurodema thaul*	Andes	semi-aquatic	restricted	LC	[68]
Leptodactylidae	*Pseudopaludicola falcipes*	Pampa	semi-aquatic	wide	LC	[25]
Microhylidae	*Dermatonotus muelleri*	Chaco/Pantanal	fossorial	wide	LC	[53]
Microhylidae	*Dermatonotus muelleri*	Chaco/Pantanal	fossorial	wide	LC	[69]
Odontophrynidae	*Odontophrynus americanus*	Patagonia	fossorial	wide	LC	[70]
Odontophrynidae	*Odontophrynus asper*	Pampa	fossorial	wide	LC	[29]
Odontophrynidae	*Odontophrynus asper*	Chaco/Pantanal	fossorial	wide	LC	[71]
Odontophrynidae	*Odontophrynus asper*	Chaco/Pantanal	fossorial	wide	LC	[46]
Odontophrynidae	*Odontophrynus cordobae*	Chaco/Pantanal	fossorial	restricted	LC	[71]

**Figure 4.**
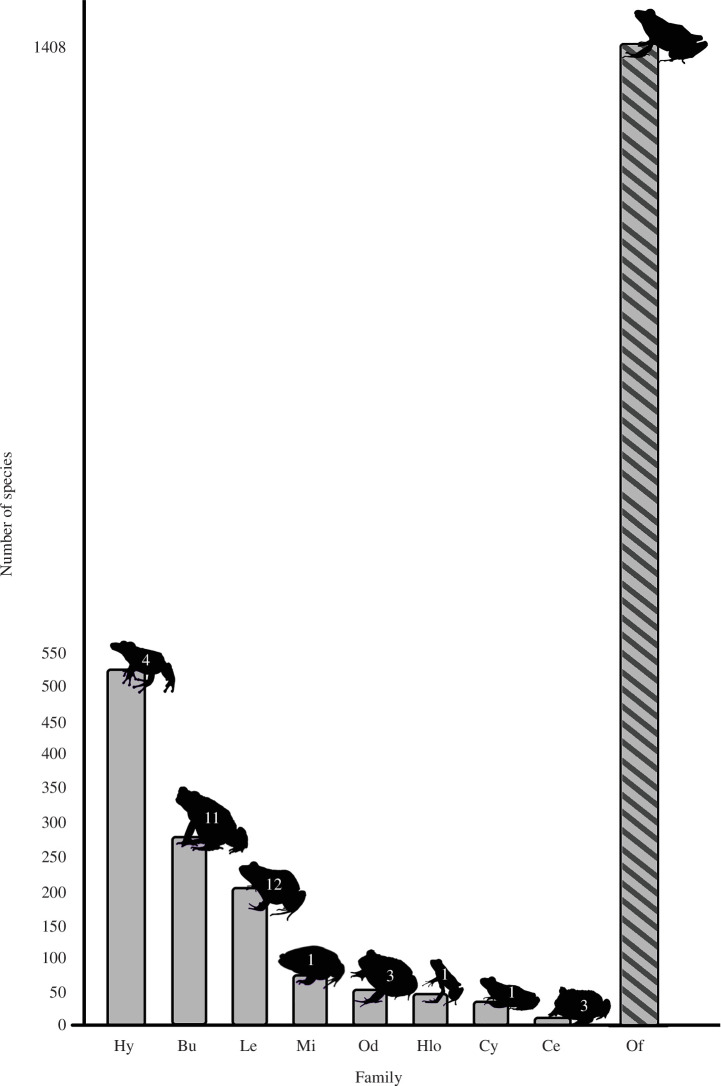
Number of anuran species per family and the number of South American species for which longevity is known (inside the silhouettes). Hy = Hylidae; Bu = Bufonidae; Le = Leptodactylidae; Mi = Microhylidae; Od = Odontophrynidae; Hlo = Hylodidae; Cy = Cycloramphidae; Ce = Ceratophryidae; Of = other South American anuran families. Gray and black hatched bar = sum of species without longevity data. The silhouettes are not to scale. Anuran families according to Vasconcelos *et al*. [37].

Out of the 24 anuran families that occur in South America, only eight (33%) had information on species’ longevity. A total of eight publications comprised longevity data on 12 species of the Leptodactylidae family (which represents 6% of the described leptodactylids). For the Bufonidae family, information on longevity was found for 11 species (4% of described species), published in 11 scientific articles. For the Hylidae family, information on longevity was found for four species (0.76% of described species), published in six papers. The families Ceratophryidae and Odontophrynidae had information for only three species each (25% and 5.66% of the described species, respectively), published in three and four papers, respectively. Cycloramphidae, Hylodidae and Microhylidae were the families with the greatest deficits in knowledge on longevity, with information for only one species of each family (2.78%, 2.17% and 1.35% of the described species, respectively), within four articles (table 3, figure 4).

**Table 3.** Table of South American species by family and number of species in these families with longevity data. Data presented in descending order of representativeness.

family	species of South America	species with data about longevity	%
Ceratophryidae	12	3	25
Leptodactylidae	200	12	6
Odontophrynidae	53	3	5.66
Bufonidae	274	11	4.01
Cycloramphidae	36	1	2.78
Hylodidae	46	1	2.17
Microhylidae	74	1	1.35
Hylidae	520	4	0.76

**Figure 5.**
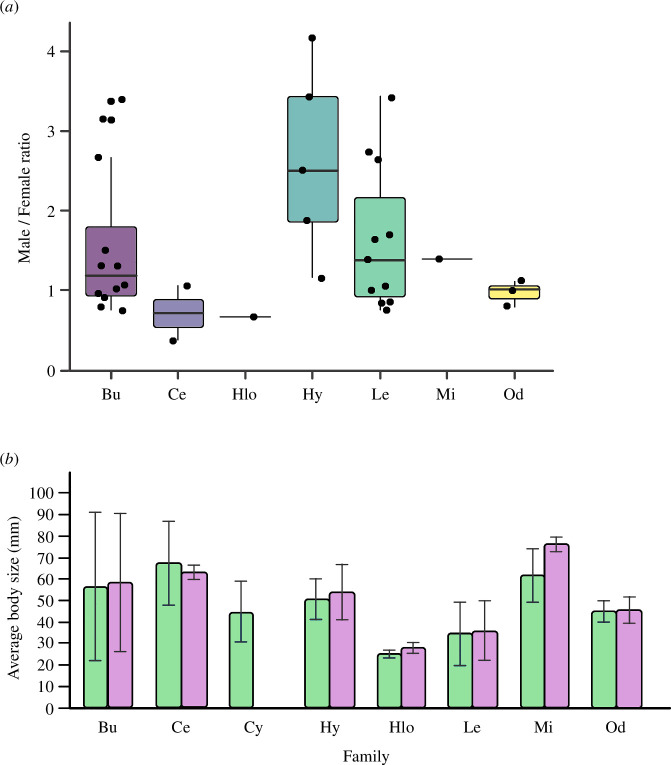
(*a*) Ratio of the sample number of males and females per family used in the studies. (*b*) Average body size of anuran species used in skeletochronology studies per family, in South America. Average body size (SVL) data of the species were taken directly from the articles and the average of the SVL found for each family was calculated. Hy = Hylidae; Bu = Bufonidae; Le = Leptodactylidae; Mi = Microhylidae; Od = Odontophrynidae; Hlo = Hylodidae; Cy = Cycloramphidae; Ce = Ceratophryidae; Gray line = standard deviation; Gray and orange hatched bar = general average size of the species.

In general, the sample size used in the studies differed between males and females, with males being more targeted for longevity studies than females (ratio >1) (electronic supplementary material, S2). When we analysed by family, we recovered those studies on species of Bufonidae, Hylidae and Leptodactylidae families typically used a larger sample number of males, while females were more used in studies on species from Ceratophryidae and Odontophrynidae families (figure 5*a*). It is worth mentioning that, of the 36 South American species on which there are longevity studies, for more than 20% of them (eight species) the longevity of females has not been investigated (electronic supplementary material, S1). The average snout-vent length (SVL) of the species for which longevity has been studied was 47.73 ± 23.93 mm for males, and 48.95 ± 24.82 mm for females, where only Bufonidae, Ceratophryidae, Hylidae and Microhylidae had an average SVL lower than 50 mm (figure 5*b*, electronic supplementary material, S1).

**Figure 6.**
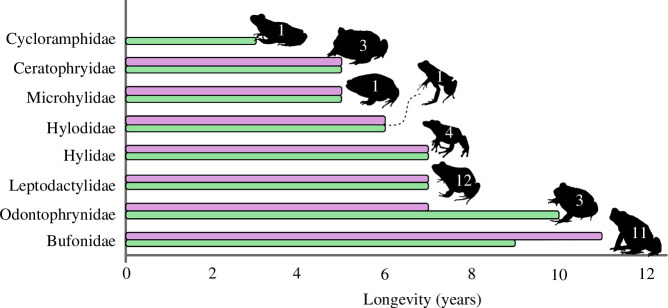
Estimated longevity (in years) for each South American anuran family using skeletochronology. Green bars = male longevity; purple bars = female longevity; the values within the silhouettes represent the number of species in each family for which longevity work has been published. The silhouettes are out of scale.

Regarding longevity, Bufonidae and Odontophrynidae are the families with the greatest longevity recorded for females (11 years) and males (10 years), respectively. (table 4). Species from the Leptodactylidae and Hylidae families had the same maximum longevity (7 years for males and females). The lowest longevity was recorded for species of the families Hylodidae (6 years for males and females), Ceratophryidae and Microhylidae (5 years for males and females). For the Cycloramphidae family, the only study provided longevity only for males (3 years) of one species (figure 6). The mean age found for the families analysed varied between 3 and 6 years (table 4; electronic supplementary material, S3), with Bufonidae presenting the highest average longevity (6 years for males and females), and Cycloramphidae the lowest (3 years for males). The average age of sexual maturity was the same for males and females in the families Hylidae, Leptodactylidae and Microhylidae (2 years), Ceratophryidae and Odontophrynidae (1 year), but it varied between males and females in the families Bufonidae and Hylodidae, where males reach sexual maturity, on average, at 2 years and females at 3 years (table 4; electronic supplementary material, S3). In general, the average reproductive potential of families varied between 2 and 5 years, with Odontophrynidae presenting the highest average reproductive potential (5 years for females and 4 for males) and males of Ceratophryidae, Leptodactylidae and Microhylidae had the lowest average reproductive potential (both 2 years) (table 4; electronic supplementary material, S3).

**Table 4.** Table of species age data. Bu = Bufonidae; Ce = Ceratophryidae; Cy = Cycloramphidae; Hy = Hylidae; Hlo = Hylodidae; Le = Leptodactylidae; Mi = Microhylidae; Od = Odontophrynidae. *n* = total sample number used. SVL = snout vent length. ♂ = male. ♀ = female. SVL expressed in mm ± s.d.. Maximum and average longevity, sexual maturity and reproductive potential expressed in years.

family	species	N	SVL		maximun longevity		mean Age		sexual maturity		reproductive potential		reference
			♂	♀	♂	♀	♂	♀	♂	♀	♂	♀	
Bu	*Atelopus lozanoi*	5	28.65	42.45	4	4	—	—	—	—	—	—	[31]
Bu	*Atelopus peruensis*	14	39.55	43.4	6	—	—	—	—	—	—	—	[31]
Bu	*Melanophryniscus atroluteus*	38	23.63 ± 1.18	25.76 ± 1.41	7	9	4	5	3	3	4	6	[45]
Bu	*Melanophryniscus atroluteus*	53	21.41 ± 1.89	22.49 ± 1.33	6	6	3	4	2	2	4	4	[46]
Bu	*Melanophryniscus devincenzii*	30	23.49 ± 1.16	27.45 ± 1.54	7	7	5	5	3	4	4	3	[45]
Bu	*Melanophryniscus krauczuki*	35	20.88 ± 1.21	23.52 ± 1.44	5	5	3	3	2	2	3	3	[45]
Bu	*Melanophryniscus moreirae*	55	23.2 ± 0.2	26.2 ± 0.2	6	6	4	5	2	3	4	3	[47]
Bu	*Rhinella achalensis*	205	57.96 ± 6.75	54.59 ± 6.8	9	11	5	4	—	—	—	—	[48]
Bu	*Rhinella arenarum*	88	82.5 ± 11.45	69.17 ± 8.13	6	8	—	—	—	—	—	—	[44]
Bu	*Rhinella arenarum*	138	100.45 ± 7.95	108.6 ± 9.6	6 y	4	3	2	1	2	5	2	[49]
Bu	*Rhinella arnarum*	114	96.34 ± 9.02	106.18 ± 5.09	5	4	2	3	1	2	3		[49]
Bu	*Rhinella arenarum*	76	90.16 ± 9.4	96.53 ± 7.73	5	5	3	3	2	2	3	3	[51]
Bu	*Rhinella diptycha*	29	118.4 ± 25.44	102.6 ± 41.55	5	4	—	—	—	—	—	—	[32]
Bu	*Rhinella ornata*	116	—	—	4	—	1	—	—	—	—	—	[52]
Bu	*Rhinella rubescens*	52	51.4 ± 21.35	45.2 ± 19.57	3	3	—	—	—	—	—	—	[32]
Ce	*Ceratophrys cranwelli*	6	88.9 ± 3.46	—	2	—	—	—	—	—	—	—	[53]
Ce	*Ceratophrys stolzmanni*	152	59.79 ± 3.67	64.87 ± 4.67	3	4	2	2	1	1	2	3	[54]
Ce	*Chacophrys pierottii*	26	51.44 ± 2.33	59.14 ± 4.16	5	5	3	4	1	1	4	4	[55]
Cy	*Thoropa miliaris*	92	43.8 ± 15.2	—	3	—	1	—	—	—	—	—	[56]
Hy	*Boana cordobae*	60	48.01 ± 4.99	51.27 ± 5.06	5	5	3	3	2	2	3	3	[57]
Hy	*Boana cordobae*	129	48.85 ± 3.32	53.61 ± 5.26	7	7	3	3	2	2	5	5	[58]
Hy	*Boana cordobae*	102	49.16 ± 3.83	52.5 ± 3.8	5	6	3	3	3	3	2	3	[51]
Hy	*Boana pulchella*	63	46.34 ± 2.97	—	5	—	3	—	2	—	3	—	[59]
Hy	*Nyctimantis siemersi*	56	69.17 ± 3.56	74.19 ± 4.14	5	5	3	4	2	3	3	2	[60]
Hy	*Scinax fuscovarius*	43	38.96 ± 4.85	37.78 ± 4.31	5	6	3	2	2	1	3	5	[46]
Hlo	*Crossodactylus schmidti*	103	25.03 ± 1.33	27.68 ± 2.26	6	6	4	4	2	3	4	3	[61]
Le	*Leptodactylus bufonius*	31	55.3 ± 1.8	56.33 ± 2	4	5	2	1	1	1	3	4	[62]
Le	*Leptodactylus latinasus*	17	33.05 ± 0.75	—	6	—	4	—	3	—	3	—	63]
Le	*Leptodactylus lati-sus*	24	32.38 ± 2.94	33.02 ± 3.15	3	2	2	1	1	1	2	1	[62]
Le	*Leptodactylus luctator*	183	65.41 ± 28.84	63.59 ± 26.72	5	5	2	2	1	1	4	4	[64]
Le	*Leptodactylus mystacinus*	18	47.65 ± 2.5	—	7 y	—	4 y	—	3 y	—	3 y	—	[63]
Le	*Physalaemus biligonigerus*	29	34.69 ± 2.44	35.27 ± 2.54	5	4	3	3	2	3	3	1	[72]
Le	*Physalaemus cuvieri*	35	26.15 ± 3.1	28.14 ± 2.7	7	7	4	3	2	2	5	5	[25]
Le	*Physalaemus fernandezae*	64	20.49 ± 0.77	22.29 ± 1.15	6	6	4	4	2	3	4	3	[66]
Le	*Physalaemus riograndensis*	22	18.01 ± 0.89	18.55 ± 2.2	5	5	3	3	1	2	4	3	[25]
Le	*Pleurodema cordobae*	50	35.69 ± 1.74	40.43 ± 3.63	4	6	3	5	3	5	2	2	[67]
Le	*Pleurodema kriegi*	41	34.41 ± 2.6	37.76 ± 1.81	4	5	3	4	3	3	2	3	[67]
Le	*Pleurodema thaul*	83	32 ± 1.04	34.3 ± 1.02	5	5	—	—	2	2	—	—	[68]
Le	*Pseudopaludicola falcipes*	35	14.22 ± 1.26	15.04 ± 0.91	4	5	3	3	1	1	3	3	[25]
Mi	*Dermatonotus muelleri*	8	52.84 ± 3.08	—	2	—	—	—	—	—	—	—	[53]
Mi	*Dermatonotus muelleri*	43	70.2 ± 2.92	75.86 ± 3.78	5	5	3	3	2	2	3	3	[69]
Od	*Odontophrynus americanus*	38	51.46 ± 4.64	52.42 ± 4.13	3	4	2	2	1	1	1	3	[70]
Od	*Odontophrynus asper*	48	41.68 ± 5.8	43.13 ± 4.8	10	7	4	4	1	1	9	6	[29]
Od	*Odontophrynus asper*	34	46.36 ± 2.58	—	6	—	4	—	2	—	4	—	[71]
Od	*Odontophrynus asper*	25	40.58 ± 4.31	39.68 ± 3.04	5	7	4	3	1	1	4	4	[46]
Od	*Odontophrynus cordobae*	34	47.2 ± 2.97	—	7	7	4	—	2	—	5	—	[71]

## 4. Discussion

Although determining the age of amphibians using the skeletochronology method has been used for more than 50 years, in South America, there are few studies using this approach. The lack of studies in the Neotropics is likely due to regional differences in research effort, as well as the considerable difficulty in accessing amphibian habitats in tropical forests, as they are characterized by dense vegetation and hot and humid climates [5]. In addition, longevity information through skeletochronology requires specific laboratory infrastructure, which includes equipment (rotating microtome and electronic microscope with coupled camera), and reagents (decalcifier, historesin and dyes) for correct histological processing of the samples. Laboratory cost is often not accessible in countries with little investment in basic research. Moreover, South America has the greatest diversity of anuran species in the world [37,40], with regions considered hotspots of biodiversity [73,74]. When exploring such diverse regions, researchers tend to focus on identifying and describing species rather than identifying parameters such as longevity.

In the last 33 years, 32 studies on the longevity of South American frogs were published, mostly between 2015 and 2019. Most of these are concentrated in the Chaco/Pantanal biogeographical region, in Argentina (22 studies) and in Brazil (seven studies), followed by Chile, Colombia, Peru and Ecuador, each with only one study published. This discrepancy in the number of publications per country can be explained because Brazil and Argentina have greater access to research funds and a higher proportion of herpetologists [75]. Furthermore, the divergence in the number of publications among biogeographic regions seems to be related to the geographic location of the research laboratories working in this line, since scientific investigations tend to concentrate near locations that offer convenient access, infrastructure and logistics [76,77].

Peng *et al*. [5] highlighted a global trend of amphibian species with terrestrial habitats are one of the most targeted for longevity studies, and this pattern also was recovered for South American anurans. In addition, our results also highlight the arboreal anuran group as the least targeted in longevity studies in South America. This can be explained because animals that use vegetation can sometimes be difficult to collect [78,79], compared with those associated with the soil, resulting in less representation of the first group in scientific collections.

Regarding threat categories, in South America there is a greater prevalence of studies on the longevity of Least Concern (LC) species (>80% of species) than those allocated to other categories, contradicting the argument that scientific research efforts are driven by global risk of extinction of a species [80,81]. In fact, Silva *et al*. [82] argue that the low probability of threatened species being associated with no or few studies show that, often, the need for conservation is overcome by more practical factors (e.g. local conservation priority, abundant and easily accessible species) when researchers need to decide which species are most appropriate for a given scientific study. Also, in general, LC species are those with a wide geographic distribution, present in high abundance across various types of habitats (including modified environments), and therefore with more individuals deposited in collections than species falling into any threat category. Unfortunately, comparison with patterns recorded in other studies is unfeasible at this moment, since we failed to find previous analysis on this subject.

Although the age composition of a population is a key demographic trait, with implications for the population dynamics of the species [27,34], South American anurans are still little investigated regarding this parameter. South America has a high diversity of anuran amphibians, which are distributed in 24 families [37]. However, only eight of them have any information about longevity. Hylidae, for example, is the second richest family in number of species [83] but presented the least known longevity of species (0.76%), following the same pattern reported by Peng *et al*. [5] in the global review. On the other hand, longevity data are available for 25% of Ceratophryidae species. This number is even more discrepant when we consider the total number of anuran species in South America. In fact, out of the more than 2623 anuran species known for South America [37], only 36 of them have available longevity data, which represents 1.29% of the species in this region.

Following the global pattern evidenced by Peng *et al*. [5], Bufonidae and Leptodactylidae families are the most studied regarding species longevity: 10 studies published with bufonids (11 species) and eight with leptodactylids (12 species). This is probably due to the ease of working with these anurans that generally have medium to large body size (e.g. [29,32,52,57,64,67]). Furthermore, the predominance of studies on Bufonids and Leptodactylids can be related to species in this group that are commonly found in peri-urban environments, facilitating the specimen collection [84]. On the other hand, there are few studies with small-bodied or hard-to-find species (e.g. [25,45,47,66], and therefore we recommend future research effort directed at species in these categories.

Regarding sampling biases, and contrary to the global standard in studies with anurans [5], our results suggest a male-biased tendency in South America. This sex-biased pattern makes it difficult to evaluate sex-specific life history strategies, such as reproductive rate and survival, in addition to making it impossible to carry out a more in-depth analysis of how sexual differences can affect ecological and evolutionary processes [43,85–87]. Analysing the male/female ratio used within families, we observed that there is a greater use of males in studies on Leptodactylidae and Hylidae, probably associated with the facility to detect calling males than females [88]. For Bufonidae and Ceratophryidae, we found a female-biased tendency, which may be related to the explosive reproductive dynamics of some species in these families, which allow for a greater collection of females during reproductive peaks [89,90]. Also, in bufonids, we find species that live associated with human dwellings, facilitating the encounter of males and females in the peri-anthropic landscape [84].

Longevity evolves in response to local abiotic and biotic factors [10]. The maximum longevity of South American anurans did not exceed 10 years for males and 11 years for females, with the families Odontophrynidae and Bufonidae having the species with the longest longevity. The average longevity varied between families from 3 to 6 years. Although the estimated maximum longevity for Odontophrynidae and Bufonidae is high when compared with other families, the average longevity of the families studied did not exceed that expected for anurans living in tropical and subtropical regions, where it normally does not exceed 9 years [91,92]. Overall, longevity within families did not vary between sexes, contrary to what is expected, since males tend to live for a shorter time than females due to the high predation pressure that they experience during the calling season [93,94]. Thus, extracting overall patterns remains challenging as only a small fraction of anurans has been studied regarding male and female longevity.

Although [5] provided a global review of studies involving amphibian longevity, information such as average age, maximum longevity, age of sexual maturity and reproductive potential of the species has not been investigated in depth. Thus, our work provides the first general information on family/species patterns for these parameters. As expected for anurans living in temperate and subtropical regions, the average age of sexual maturity was 2 years for males and females from the families Hylidae, Leptodactylidae and Microhylidae, as well as for males from the families Bufonidae and Hylodidae. This pattern occurs because, generally, anurans that live in these regions experience a well-marked climatic seasonality, determined by the variation in photoperiod and air temperature [25,29,30,61,66,67,95]. In Ceratophryidae and Odontophrynidae, males and females follow the pattern proposed for anurans that live in tropical regions, but which has already been demonstrated for anurans from subtropical regions (refer to [25,29,64–66], reaching sexual maturity at just 1 year old [33]). The anticipation of sexual maturity in these species may be the result of factors such as predation pressure, female competition or growth rate [96–98], as well as the reproductive strategies adopted by them (i.e. explosive reproduction) [89,99].

The average reproductive potential of the families varied between 2 and 5 years, with males of Ceratophryidae, Leptodactylidae and Microhylidae showing the lowest average reproductive potential (only 2 years). This low average reproductive potential in ceratoprhyids, leptodactylids and microhylids is unexpected, as it implies that males from these families have a low reproductive life expectancy [65]. Furthermore, we emphasize that the low reproductive potential is even more worrying when it comes to the Ceratophryidae family, which has members with ephemeral demography, with explosive reproduction [89] and threatened with extinction (refer to [54].

Our results suggest that efforts for future studies of longevity of South American anurans should focus mainly on families few or not yet studied (e.g. Craugastoridae, Hylidae, Centrolenidae and Dendrobatidae), with high species diversity, including small-bodied ones. Also, we suggest that future work should also be aimed at species with terrestrial habitats, with more restricted geographical distribution, as well as for species at risk of extinction. Furthermore, we suggest that sampling should be carried out in other countries than Argentina and Brazil, in order to expand the representation of different evolutionary lineages along the biogeographical units of South America. Moreover, considering the basic laboratory infrastructure demands for longevity studies and the low scientific investment in South American countries, we suggest that researchers who are interested in studying the longevity of South American anurans seek collaboration with more experienced South American researchers in the area (e.g. Argentinian and/or Brazilian researchers), or even with researchers from Europe, North America and China [5].

## Data Availability

This article has no additional data. Supplementary material is available online [100].
